# Intranasal Infection of Ferrets with SARS-CoV-2 as a Model for Asymptomatic Human Infection

**DOI:** 10.3390/v13010113

**Published:** 2021-01-15

**Authors:** Helen E. Everett, Fabian Z. X. Lean, Alexander M. P. Byrne, Pauline M. van Diemen, Shelley Rhodes, Joe James, Benjamin Mollett, Vivien J. Coward, Paul Skinner, Caroline J. Warren, Kevin R. Bewley, Samantha Watson, Shellene Hurley, Kathryn A. Ryan, Yper Hall, Hugh Simmons, Alejandro Núñez, Miles W. Carroll, Ian H. Brown, Sharon M. Brookes

**Affiliations:** 1 Virology Department, Animal and Plant Health Agency, New Haw, Addlestone, Surrey KT15 3NB, UK; Alexander.Byrne@apha.gov.uk (A.M.P.B.); PaulineM.vanDiemen@apha.gov.uk (P.M.v.D.); Joe.James@apha.gov.uk (J.J.); Benjamin.Mollett@apha.gov.uk (B.M.); Vivien.Coward@apha.gov.uk (V.J.C.); Paul.Skinner@apha.gov.uk (P.S.); Caroline.Warren@apha.gov.uk (C.J.W.); Ian.Brown@apha.gov.uk (I.H.B.); Sharon.Brookes@apha.gov.uk (S.M.B.); 2 Pathology and Animal Sciences Department, Animal and Plant Health Agency, New Haw, Addlestone, Surrey KT15 3NB, UK; fabian.lean@apha.gov.uk (F.Z.X.L.); Samantha.Watson@apha.gov.uk (S.W.); shellene.hurley@apha.gov.uk (S.H.); Hugh.Simmons@apha.gov.uk (H.S.); alejandro.nunez@apha.gov.uk (A.N.); 3 Bacteriology Department, Animal and Plant Health Agency, New Haw, Addlestone, Surrey KT15 3NB, UK; shelley.rhodes@apha.gov.uk; 4 National Infection Service, Public Health England (PHE), Porton Down, Salisbury, Wiltshire SP4 0JG, UK; Kevin.Bewley@phe.gov.uk (K.R.B.); Kathryn.Ryan@phe.gov.uk (K.A.R.); Yper.Hall@phe.gov.uk (Y.H.); Miles.Carroll@phe.gov.uk (M.W.C.); 5 Nuffield Department of Medicine, Oxford University, Oxford OX1 3SY, UK

**Keywords:** SARS-CoV-2, ferret, Y453F, olfactory neuronal cells

## Abstract

Ferrets were experimentally inoculated with SARS-CoV-2 (severe acute respiratory syndrome (SARS)-related coronavirus 2) to assess infection dynamics and host response. During the resulting subclinical infection, viral RNA was monitored between 2 and 21 days post-inoculation (dpi), and reached a peak in the upper respiratory cavity between 4 and 6 dpi. Viral genomic sequence analysis in samples from three animals identified the Y453F nucleotide substitution relative to the inoculum. Viral RNA was also detected in environmental samples, specifically in swabs of ferret fur. Microscopy analysis revealed viral protein and RNA in upper respiratory tract tissues, notably in cells of the respiratory and olfactory mucosae of the nasal turbinates, including olfactory neuronal cells. Antibody responses to the spike and nucleoprotein were detected from 21 dpi, but virus-neutralizing activity was low. A second intranasal inoculation (re-exposure) of two ferrets after a 17-day interval did not produce re-initiation of viral RNA shedding, but did amplify the humoral response in one animal. Therefore, ferrets can be experimentally infected with SARS-CoV-2 to model human asymptomatic infection.

## 1. Introduction

In December 2019, clinical cases of pneumonia of unknown aetiology were first reported in Wuhan, Hubei province in Central China. Metagenomic sequencing revealed the causative agent to be a novel, severe acute respiratory syndrome (SARS)-related coronavirus, designated SARS-CoV-2 [1]. One year later, the number of reported cases of human infection continues to grow globally [2], showing the high transmissibility of this human pandemic coronavirus of zoonotic origin. The majority of cases (80%) are asymptomatic or have mild disease [3], presenting a challenge for monitoring virus infection and transmissibility and for determining appropriate public health policy.

Coronaviruses have been identified in diverse mammalian species, with two zoonotic strains being of substantial public health concern this century; SARS-CoV in 2002 and Middle Eastern respiratory syndrome coronavirus (MERS) in 2012 [4]. SARS-CoV-2 is the most recent coronavirus to have emerged and, based on sequence identity, is likely to have originated from bats, although the possible involvement of an intermediate host species is currently unknown [5]. Similar to SARS-CoV, the infection of susceptible cells relies on the binding of the SARS-CoV-2 spike protein to angiotensin converting enzyme type 2 (ACE2) receptors and is activated by the enzyme TMPRSS2 [6]. During replication, the viral spike (S) envelope glycoprotein and the nucleoprotein (NP) are highly transcribed [7]. Through experimental infection studies, SARS-CoV-2 has been shown to infect non-human primates, cats, ferrets, bats, hamsters and tree shrews but does not productively infect pigs, dogs or poultry [8,9,10,11,12,13,14,15,16,17,18,19]. Sporadic infections of domestic cats and dogs, as well as large captive felids and farmed mink [20,21,22,23,24] have been reported and are thought to be reverse-zoonoses.

Understanding the pathogenesis of SARS-CoV-2 in an animal model for human disease [25] is important to gain insight into disease dynamics and for the development of therapeutic interventions. The ferret has been conventionally used in animal models for respiratory pathogen infections, including influenza and previously with SARS-CoV [26]. Recent reports of several ferret studies [8,9,11,17,27] and this study provide further insight into virus–host interactions in this animal model.

## 2. Materials and Methods

### 2.1. Cells and Viruses

Vero E6 cells were cultured in Dulbecco’s modified Eagle’s medium (DMEM) supplemented with 10% heat-inactivated fetal calf serum (FCS), HEPES, sodium bicarbonate, 100 units/mL penicillin and 1000 µg/mL streptomycin (Gibco, Dartford, UK). The SARS-CoV-2 inoculum of strain SARS-CoV-2/human/Australia/VIC01/2020 (GISAID accession number EPI_ISL_406844) was propagated in Vero/hSLAM cells (ECACC 04091501) and supplied by Public Health England [27]. The phylogenetically identical isolate SARS-CoV-2/human/Italy/LAZ-INMI1-isl/2020 (GISAID accession number EPI_ISL_410545) was provided by the Italian Institute for Infectious Diseases (INMI) through the European Virus Archive GLOBAL (EVA-GLOBAL), propagated in Vero E6 cells and used for downstream serological analysis. Virus titration and isolation from clinical samples was based on cytopathic effect in Vero E6 cells at 5 dpi. The tissue culture 50% infectious dose (TCID_50_) was calculated according to the method of Spearman–Karber [28,29,30].

### 2.2. In Vivo Study

The ferret in vivo study was conducted in accordance with UK Home Office regulations under the Animal (Scientific Procedures) Act 1986 (ASPA) with study PP3405816/1/001 approved on 30 April, 2020 by the Animal Welfare and Ethical Review Body (AWERB) of the Animal and Plant Health Agency and was reported according to the ARRIVE guidelines [31]. Twelve female ferrets approximately 5 months of age were housed in two groups of six. General anaesthesia was performed for inoculation and sample collection, with 4.5% isoflurane (Zoetis, Leatherhead, UK) chamber induction followed by a single subcutaneous injection of medetomidine (0.04 mg/kg, Vetoquinol, Towcester, UK) and butorphanol (0.1 mg/kg, MSD Animal Health, Milton Keynes, UK). Reversal of medetomidine sedation was achieved with a subcutaneous injection of atipamezole hydrochloride (0.4 mg/kg, Vetoquinol). Ferrets were inoculated by intranasal (IN) instillation with 1.2 × 10^6^ TCID_50_/mL of SARS-CoV-2/Australia/VIC01/2020 [32] delivered in 0.5 mL per nostril. At 21 days post-inoculation (dpi), two ferrets were re-challenged IN with 2.0 × 10^6^ TCID_50_/mL of the same inoculum. Clinical monitoring and sampling were done as described (Table 1).

Weight, temperature (subcutaneous Biothermal Identichip^®^, Destron Fearing, Dallas, TX, USA) and clinical signs were monitored daily. Clinical samples of nasal washes in Dulbecco’s PBS (DPBS, Gibco) as well as oro-pharyngeal (throat) and rectal swabs (MWE, Corsham, UK) were obtained prior to infection and on 2, 4, 6, 8, 10, 14, 19 and 21 dpi. At the same time, environmental samples of food, water and swabs (Copan Diagnostics, Murrieta, CA, USA) of the metal cage surface and ferret fur coat, along the dorsal midline were collected. Samples were stored at 4 °C until processing and archived at –80 °C. Blood samples (clotted and EDTA anticoagulated) were taken from the jugular vein or cranial vena cava prior to infection and on 4, 8, 14 and 21 dpi. Necropsies of two ferrets were performed on each of 3, 5, 7, 14 and 21 dpi as well as, for two ferrets, 7 days after re-challenge (24 dpi). Bronchoalveolar lavage (BAL) was performed on the left lung lobe after euthanasia. A comprehensive panel of respiratory, gastrointestinal and lymphoid tissues were taken for virological and pathological analyses. Tissue samples for virological analysis were stored at –80 °C in L-15 Leibovitz medium containing 1% (*v*/*v*) FCS, 100 units/mL penicillin and 1000 µg/mL streptomycin (all Gibco). Samples for pathological examination were fixed in 10% neutral buffered formalin at room temperature.

### 2.3. Viral RNA Isolation and Real-Time RT-qPCR

Swabs and tissue samples were suspended in L-15 Leibovitz medium containing 1% (*v*/*v*) FCS, 100 units/mL penicillin and 1000 µg/mL streptomycin (all Gibco). Total RNA was extracted from all samples using a QIAmp Viral RNA Biorobot Kit (Qiagen, Manchester, UK). Viral RNA was detected using the SARS-CoV-2 E gene real-time RT-qPCR [33]. The reported primer and probes sequences used were E_Sarbeco_F 5′-ACAGGTACGTTAATAGTTAATAGCGT-3′, E_Sarbeco_R 5′-ATATTGCAGCAGTACGCACACA-3′ and E_Sarbeco_P1 5′-FAM-ACACTAGCCATCCTTACTGCGCTTCG-BBQ (Eurogentec, Seraing, Belgium). Viral RNA quantity is expressed as relative equivalent units (REU) of RNA using a standard 10-fold dilution series of RNA purified from the same batch of virus, of known TCID_50_ titre, used for the inoculation. A virus stock diluted in standard tissue culture medium was used to minimize potential confounding effects caused by the sample matrix, should it contain substances inhibitory to either PCR or virus titration. Viral genome copies were quantified using a tenfold dilution series of an Ultramer DNA oligonucleotide equivalent to 120 bp of the SARS-CoV-2 E-gene (nCoV-E-Sarbeco control plasmid) with sequence 5′-GAGACAGGTACGTTAATAGTTAATAGCGTACTTCTTTTTCTTGCTTTCGTGGTATTCTTGCTAGTTACACTAGCCATCCTTACTGCGCTTCGATTGTGTGCGTACTGCTGCAATATTGTT. There was direct correlation between REU and genome copy standard lines (Supplemental Figure S1) and a lower limit of quantification (LLoQ) was set at 0.4 REU/mL. Although REU and genome copy number correlate with the amount of viral RNA present and not infectivity, it may be inferred from the linear relationship with the dilution series that these measures are proportional to the amount of infectious virus present.

### 2.4. Whole Genome Sequencing

Viral RNA was extracted manually using the QIAamp Viral RNA Mini Kit (Qiagen) from clinical samples according to the manufacturer’s instructions but without carrier RNA and eluted in 25 µL nuclease-free water. For clinical samples, viral RNA was then used to generate double-stranded cDNA using sequence-independent single-primer amplification (SISPA) [34] and purified using AMPure beads (Beckman Coulter, Brea, CA, USA). Viral first-strand cDNA synthesis was performed using SuperScript IV (Invitrogen, Carlsbad, USA) and second-strand synthesis using the NEBNext Ultra II nondirectional RNA second-strand synthesis module (New England Biolabs, Ipswich, MA, USA). Library preparation was performed using the Nextera DNA Library Prep Kit (Illumina, Cambridge, MA, USA) and sequenced using the NextSeq System (Illumina). All kits were used as per the manufacturer’s instructions. Paired-end Illumina reads were assembled using a custom reference-guided alignment script (https://github.com/AMPByrne/WGS/blob/master/RefGuidedAlignment.sh) using the inoculum reference sequence. Sequence outputs were aligned using MAFFT version 7.427 [35], visualized using MEGA-X [36] and sequence variants determined using flutile (https://github.com/flu-crew/flutile).

### 2.5. Serology

SARS-CoV-2 antibody levels were evaluated in heat-inactivated serum samples. Antibody titres to His-tag recombinant viral proteins spike subunit 1 (S1) (REC31828-100) and nucleoprotein (NP) (REC31812-100) (gifts from The Native Antigen Company, Oxford, UK) were determined by direct ELISA. Antigen-coated ELISA plate wells (Nunc Maxisorp, Thermo Scientific, Rockford, IL, USA), or control wells with no antigen, were blocked with 20% soya milk in PBS and washed with PBS/0.1% Tween-20 before adding serum samples diluted in 20% soya milk/PBS/0.05% Tween-20 (S1 1:400, NP 1:50). Plates were then incubated with Protein–AG–HRP (catalogue no. 32490, Thermo Scientific, diluted 1:20,000 in 5% soya milk/PBS/0.05% Tween-20). Antibody binding was detected with TMB (catalogue no. T0440, Sigma-Aldrich, St Louis, MO, USA), and the reaction was then stopped with 0.5 M H_2_SO_4_ and evaluated at OD 450 nm. NP ELISA data were analysed by subtracting the no-antigen control well from the NP antigen well for each sample, to account for the higher nonspecific binding in the NP ELISA as a result of the higher concentrations of serum used.

To determine virus-neutralizing antibody titre, doubling dilutions of serum were mixed with an equal volume of 100 TCID_50_ units of SARS-CoV-2/human/Italy/LAZ-INMI1-isl/2020 and incubated at 37 °C for one hour. Following incubation, 50 µL of each dilution was transferred per well into 96-well plates containing 90% confluent Vero E6 monolayers. Following a second 1 h incubation at 37 °C and 5% CO_2_, plates were overlaid with DMEM (Gibco) without serum (WOS) and incubated for five days. The cells were examined for cytopathic effect (CPE) using an inverted light microscope, and the neutralising antibody titre was calculated according to the method of Spearman–Karber [28,29,30] and expressed as 50% inhibitory concentration (IC_50_).

### 2.6. Histopathology, Immunohistochemistry and In Situ Hybridisation

Formalin-fixed tissues were processed for routine histopathology ([37]). Four-micrometre-thick sections were dewaxed and rehydrated through xylene and graded alcohol for immunohistochemistry (IHC) or *in situ* hybridisation (ISH). For IHC, tissue sections were quenched for endogenous peroxidase with 3% methanol/hydrogen peroxide, blocked with goat serum and epitope unmasking was accomplished using pH 9 retrieval buffer (Dako, Glostrup, Denmark) or Protease XXIV (Sigma-Aldrich). This was followed by incubation with rabbit monoclonal anti-S or rabbit polyclonal anti-NP antibody (Sinobiological, Beijing, China) and anti-rabbit ENVISION^TM^ polymer (Dako). The signal was developed using 3,3-diaminobenzidine (DAB) (Sigma-Aldrich), and tissues were counterstained in Mayer’s haematoxylin (Leica, Milton Keynes, United Kingdom. Sections were washed with Tris-buffered saline between incubations. 

ISH used twenty pairs of double Z RNA probes targeting the S gene (V-nCoV2019-5, catalogue no. 848569, ACD, California, USA) with the RNAScope^®^ 2.5 HD Brown Detection Kit (ACD) as per the manufacturer’s instructions. Tissues were dewaxed and hydrated through xylene and alcohol, respectively, and treated with RNAscope^®^ hydrogen peroxide with heat-mediated retrieval used the Target Retrieval Solution and Protease Plus (all ACD). Tissue sections were then hybridised with RNA probes, followed by amplification with Hybridise Amp (ACD), and the signal was then developed with DAB and counter-stained with Mayer’s haematoxylin (Leica). Sections were washed with 1× wash buffer (ACD) between incubations. De-hydrated sections were then mounted with glass coverslips using dibutyl phthalate xylene or Cytoseal (ACD). Formalin-fixed, paraffin-embedded SARS-CoV-2/human/Italy/LAZ-INMI1-isl/2020-infected cell pellet sections were used as a positive control. Separate serial sections of tissue were stained with haematoxylin and eosin for histopathological evaluation.

### 2.7. Statistical Analysis

Graph and statistical analysis was performed with Graphpad Prism 7. ELISA statistical analysis used a Wilcoxon matched pairs, 2-tailed test.

## 3. Results

**Figure 1:**
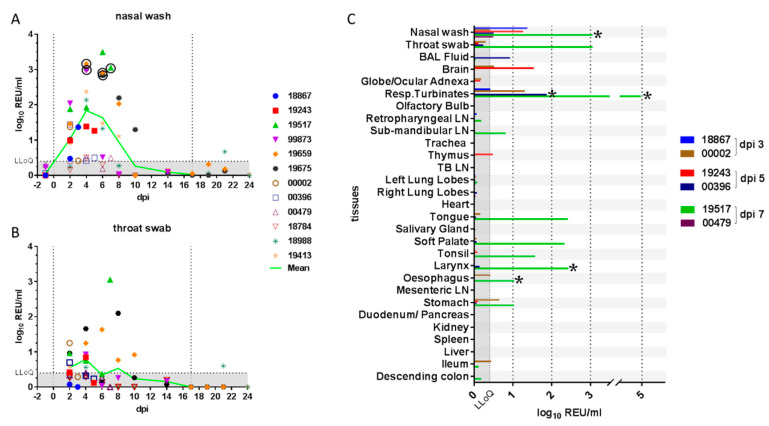
Quantification of viral RNA in clinical samples. Viral RNA quantified in (**A**) nasal wash and (**B**) throat swab samples is shown as relative equivalent units (REU) based on a viral RNA standard dilution series prepared from a stock of known titre/mL. Grey shading denotes the area below the LLoQ. Vertical lines indicate the days of inoculation and re-challenge. Viral RNA (**C**) was also quantified in tissues from 3, 5 and 7 days post-inoculation (dpi). Presence of infectious virus was verified by re-isolation in selected samples, as indicated by a circle or asterisk (*). Abbreviations: LLoQ, lower limit of quantification; BAL, bronchoalveolar lavage; Resp., respiratory; LN, lymph node; TB, tracheobronchial.

Daily monitoring of clinical parameters, including weight, temperature and respiratory signs, showed that ferrets exhibited mild or no clinical signs for the duration of the study. No weight loss was recorded outside of the normal range (5%), and temperatures remained within the normal range (maximum +1.2 °C relative to baseline body temperature), except in the case of two different ferrets that showed temperatures of +1.5 °C for single days (Supplemental Figure S2). Longitudinal samples (Table 1) were monitored for virological and immunological parameters. Viral RNA in clinical samples was quantified by real-time RT-qPCR targeting a SARS-CoV-2 E gene amplicon [33] and expressed as relative equivalent units (REU) correlated to infectious viral titre in a standard stock propagated in tissue culture. REU also correlated with viral genome copies (Supplemental Figure S1). Shedding of viral RNA, quantified in nasal washes, varied substantially between individual ferrets and reached a peak between 4 and 6 dpi, declined by 10 dpi and was below the LLoQ of the assay by 14 dpi (Figure 1A).

High levels of nasal shedding (~2.5–3 log_10_ REU/mL) were detected in four of the twelve ferrets (33%), while for two ferrets (17%), the shedding over time remained near LLoQ for the assay. Viral RNA detected in throat swabs showed the same trend as the nasal wash samples, but amounts were approximately 10-fold lower (Figure 1B). Viral RNA was also detected in rectal swabs at low levels approaching the assay LLoQ in two of the remaining ten (20%) ferrets at 6 dpi (data not shown). One of the two ferrets that were re-challenged showed some evidence of productive re-infection as viral shedding was detected above the LLoQ, on a single day, although this shedding was considerably lower than that observed following the initial infection. Infectious virus was successfully isolated from selected nasal wash samples with high levels of viral RNA (Figure 1A and Table 2).

Whole genome sequencing (WGS) analysis of viral RNA in upper respiratory tract samples from three animals identified four nonsynonymous nucleotide substitutions relative to the inoculum. The amino acid changes in the ORF1ab-encoded protein, A1670E and F1925C, were both found in samples from two of three animals, whilst the L3606F polymorphism was identified in all three animals. A single polymorphism, Y453F, was identified in the spike protein in samples from all three animals.

Viral RNA was quantified by real-time RT-qPCR in all tissues from animals necropsied at 3, 5 and 7 dpi (Figure 1C) and in selected tissues from all other ferrets necropsied on 14, 21 and 24 dpi (data not shown). Viral RNA was most abundant in tissues of the upper respiratory tract (respiratory turbinates) or oro-pharynx (soft palate, tonsil, tongue and larynx) and primarily at 7 dpi. This was immediately after the peak nasal shedding of viral RNA at 4–6 dpi (Figure 1A,B). Lower levels of viral RNA were also detected in the gastrointestinal tract (oesophagus and stomach), although this could have been due to ingestion of viral particles. No viral RNA was detected in the spleen, liver, heart, trachea, cranial lung lobes or serum from any animal (data not shown). However, low levels of viral RNA were detected in BAL fluid from the lung of one ferret on 5 dpi. The significance of viral RNA detection in two brain samples is unclear. Infectious virus was successfully isolated from selected tissue samples (Figure 1C and Table 2) but was not titrated because of practical difficulties in comparing results between different and complex sample matrixes.

Lesions were not detected on gross necropsy examination, and there was no significant increase in lung weight (not shown). Although viral RNA and antigen were not detected in the lungs by histological methods, the lungs appeared to be variably congested with occasional bronchiolitis. Systematic examination of tissues by histopathology revealed no marked changes. In the nasal turbinates, mucosal epithelial cells appeared to be uniform in shape and cilia were present, with rare presence of intra-epithelial neutrophils and apoptotic bodies. Moderate periportal lymphoplasmacytic hepatitis was observed in the liver of all ferrets, but metagenomic analysis did not reveal the presence any coronavirus sequences.

**Figure 2:**
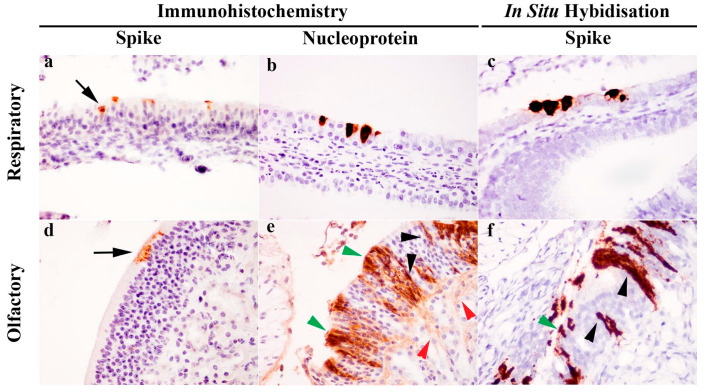
Detection of severe acute respiratory syndrome (SARS)-related coronavirus (SARS-CoV-2) in the respiratory and olfactory mucosa of infected ferrets by immunohistochemistry (IHC) and in situ hybridisation (ISH). IHC labelling detected spike antigen (**a**,**d**) in the apical cytoplasm of epithelial cells (black arrow), whereas nucleoprotein labelling (**b**,**e**) was ubiquitous throughout the cytoplasm. In the olfactory mucosa (**e**), nucleoprotein was present in various cell populations identified by morphology, including sustentacular cells (green arrowhead), olfactory neuronal cells (black arrowhead) and olfactory nerve fibres (red arrowhead). S gene ISH also revealed the presence of viral RNA in both the respiratory (**c**) and olfactory mucosa (**f**), and in the latter case, labelling was again identified in the sustentacular cells (green arrowhead) and olfactory neuronal cells (black arrowhead). Images taken with 400× objective.

Immunohistochemical (IHC) detection of viral S or NP antigen distribution in tissues revealed presence of viral antigens only within the respiratory and olfactory epithelium of the nasal cavity. At the cellular level, S antigens were localized to the apical aspect of the respiratory and olfactory epithelium (Figure 2a,d). In contrast, NP labelling was ubiquitous throughout the cytoplasm of epithelial cells (Figure 2b,e). In the olfactory epithelium (Figure 2e), the immunopositive cells had morphology suggestive of olfactory neuronal cells, in which chromogens outlined the dendrite, cell body and axon, sustentacular cells and also the neuronal tract within the lamina propria of the olfactory epithelium. Further evaluation using S gene ISH (RNAScope^®^) also confirmed this observation (Figure 2c,f). No viral RNA (Figure 1C) or viral antigen (IHC, not shown) were detected in the olfactory bulb.

**Figure 3:**
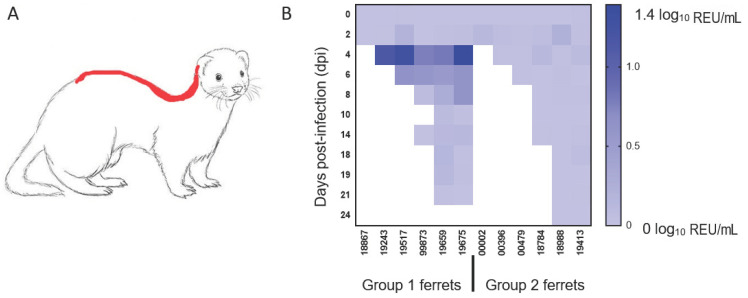
Detection of viral RNA on ferret fur. Diagram delineating in red (**A**) the sampling location for the ferret fur along the dorsal midline and (**B**) the quantification of viral RNA detected on the fur of both groups of animals on the indicated days post-infection (dpi). The shading from light blue to dark blue indicates a greater amount of viral RNA detected (log_10_ relative equivalent units (REU)/mL).

Presence of viral RNA in the environment was evaluated by analysing samples taken from food and water as well as swabs of metal cage surfaces and the fur of ferrets swabbed along the dorsal midline, a common mutual grooming location (Figure 3A). Viral RNA was not detected in food, water or swabs of the cage surfaces but was detectable in fur swab samples taken at 4 and 6 dpi, corresponding to the time of peak virus shedding (Figure 3B). This observation mainly applied to one group, which included three animals (3/6, 50%) with high levels of nasal shedding. However, no infectious virus could be re-isolated from any of the fur swab samples with low abundance of viral RNA detected by the more sensitive real-time RT-qPCR approach.

**Figure 4:**
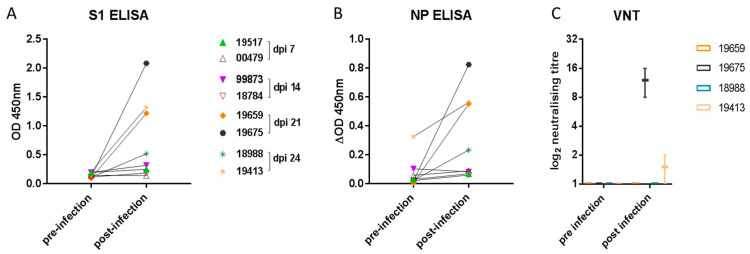
Ferret antibody response. Specific antibody elicited to (**A**) the spike (S1) glycoprotein or (**B**) nucleoprotein (NP) was detected in samples taken from two ferrets on 21 and on 24 dpi. Each data point represents a sample from one of eight ferrets pre- and post-inoculation. The graphs show the mean OD 450 nm values from two independent ELISA assays (which did not significantly differ). Neutralizing antibody (**C**) was detected in samples obtained from single ferrets on 21 and 24 dpi. The graph shows the log_2_ box-and-whisker values from two independent virus-neutralizing titre (VNT) determinations.

To determine humoral immune responses, spike protein subunit 1 (S1) and NP ELISA as well as virus neutralization assays were conducted using serum samples collected prior to infection and on 4, 8, and 14 dpi as well as at the end of the study on 21 or 24 dpi (7 days following re-challenge). Both S1- and NP-specific antibody levels increased significantly (Figure 4A,B) compared with baseline in the four ferrets sampled on 21 and 24 dpi (*p* = 0.0156 for both S1 and NP). There was no increase in antibody levels in samples from 7 or 14 dpi. Due to higher nonspecific binding in the NP ELISA, the data were analysed over a smaller OD range by subtracting the no-antigen control well from the NP antigen well for each sample. Mean background OD values for the NP ELISA were as follows: pre-infection: 0.1708 (range 0.1325–0.2035), and post-infection: 0.1884 (range 0.1085–0.2645). Of the four ferrets with detectable S1 and NP antibody by ELISA, only two of four had low neutralizing antibody titres (Figure 4C).

## 4. Discussion

This study demonstrates that ferrets can be experimentally infected with SARS-CoV-2 via the intranasal route, similar to other reports [8,9,11,17,27], with the overall infection profile resembling asymptomatic and mild clinical presentation in humans [3]. Ferrets did not exhibit overt clinical signs in this or other studies [9,17], although mild clinical signs following infection have also been reported [8,27]. Intranasal challenge of ferrets with SARS-CoV-2 resulted in productive infection within the nasal turbinate mucosae, as shown by histology, and the detection of viral RNA in nasal wash and throat swab samples, indicating localised shedding of virus into the nasal and oral cavity. Presence of infectious virus was confirmed by virus re-isolation from selected nasal wash and other upper respiratory tract samples with high abundance of viral RNA. The levels of viral RNA and ability to detect infectious virus varied between individual ferret samples, as was observed in other studies [8,9,11,17,27], and may be due to the relative ease of inactivating viral infectivity while retaining the ability to detect viral RNA in individual samples. Intranasal re-challenge of ferrets did not result in a sustained productive infection, as observed in another study [27] and also reported following infection of rhesus macaques [38]. Low levels of viral RNA were detected within the gastrointestinal tract, as also shown in other studies [8,9,11,17,27]. This finding may indicate productive infection of the gut or possibly resulted from ingestion of viral particles present in the oro-nasal cavity.

Currently, there is limited evidence of lower respiratory tract infection of ferrets with SARS-CoV-2. In this study, only low levels of viral RNA were detected in the cells isolated from the bronchoalveolar lavage of one ferret. While viral RNA or antigens were detected in the lungs in some studies [9,17,27], recovery of low levels of infectious virus has only been reported in one study [8]. To determine whether the lower respiratory tract of ferrets could support SARS-CoV-2 infection, Shi and colleagues also performed intratracheal challenge, but failed to show productive infection in the ferret lung [9]. These findings contrast with earlier studies of SARS-CoV infection in ferrets, which demonstrated dissemination of virus in the lungs [26] and associated diffuse alveolar damage resembling human SARS-like pathology. The ability of SARS-CoV to infect the lower respiratory tract was thought to be linked to ferret ACE2 distribution in type II pneumocytes and tracheobronchial submucosal glands [39], despite the low predicted binding affinity for ACE2 [40]. Although SARS-CoV-2 binds to the same host cell receptor, the differences between SARS-CoV and SARS-CoV-2 infection in ferrets could be caused by other unknown factors.

Immunohistochemical analysis revealed, for the first time, SARS-CoV-2 infection in cells of the ferret olfactory mucosae and associated neuronal tissue. Similarly, infection of olfactory neuronal cells in the golden Syrian hamster [10] was recently reported. These findings may provide insight into a possible origin of anosmia experienced in some cases of SARS-CoV-2, which is recognized as an important clinical indicator for early detection of infection [41]. In contrast to other upper respiratory viral infections, anosmia associated with SARS-CoV-2 infection can occur in the absence of clinical disease in the nasal passage [42] and usually early in the course of infection or in mildly affected or asymptomatic patients [43]. Human autopsy results have also revealed virus replication in olfactory neuronal cells, with low level of invasion across the cribriform plate into the olfactory bulb within the cranium, in a small number of cases with severe COVID-19 [44], although the relatedness of these findings to less severe clinical cases is unknown. Therefore, the significance of low levels of viral RNA detected in the brain tissue of two ferrets in the present and another study [17] modelling mild or asymptomatic infection is not known, as virus isolation or detection of antigen by IHC was not successful.

The challenge strain employed in this and another study [27] was isolated from an Australian case associated with travel to China early in the pandemic [32]. This strain is comparable to SARS-CoV-2 strains isolated in China [9], South Korea [8] and Germany [11,17] that were used in other experimental ferret infection studies. Whole genome sequencing analysis of single samples from the upper respiratory tracts of three ferrets revealed, in all samples, a L3606F polymorphism in the ORF1ab-encoded protein that has been attributed to a basal clade of SARS-CoV-2 isolates that emerged in China in January 2020 [45], and was also identified sporadically in three of five clusters in the Netherlands [23]. Another single nucleotide polymorphism corresponded to the spike protein Y453F variant that has emerged in mink in the Netherlands [23], as well as being present in the Cluster 5 variant of SARS-CoV-2 isolated from mink in Denmark [22] and in isolated human clinical cases. However, none of the other designated Cluster 5 mutations were identified in the samples we analysed. The Y453F polymorphism in the S protein has been identified in in vitro assays as a potential mutant that can escape neutralization by some therapeutic monoclonal antibodies [46]. Three-dimensional protein structure analysis has also predicted that this mutation may significantly reduce the binding affinity of neutralizing monoclonal antibodies [47] and increase the viral affinity for the human ACE2 receptor [48]. Although the SARS-CoV-2 spike protein from human isolates is predicted to have low affinity for the mink and ferret ACE2 receptors [40,49], the emergence of the Y453F variant in both field and experimental infection of mink and ferrets may indicate that this mutation promotes a functional interaction between virus spike protein and mink or ferret ACE2 receptors. In our study, the D614G polymorphism that has become globally dominant in humans was not detected. This polymorphism has been associated with increased virus entry into host cells [50] and infectivity [51]. In the hamster model, the D614G mutation significantly accelerated droplet transmission during the initial stages of infection [52], whilst in ferrets, it provided an advantage for viral replication and transmission but did not alter pathogenicity [53]. Cross-neutralization assays indicate that this mutation is not predicted to adversely impact vaccine efficacy [54]. Further work is needed to assess the impact of spike protein mutations on pathogenesis, host range and transmission of SARS-CoV-2 as well as on the host immune response.

SARS-CoV-2-infected ferrets were able to mount a humoral immune response such that virus-specific antibodies could be detected after 21 dpi. Antibody levels directed against the S1 antigen were higher in comparison with those of the NP antigen, a finding that could reflect the greater immunogenicity of the viral S envelope protein. A low neutralizing antibody response was only detected in two of four ferrets. Other studies reported neutralizing titres ranging from 1:8 to 1:1024 [8,9,17,27]. Similarly, clinical reports suggest that IgG levels can be significantly lower in asymptomatic relative to symptomatic patients [55] and the viral neutralizing antibody response in convalescent patients can be low [56].

The ferret model has also been used to assess SARS-CoV-2 dissemination, and both the airborne and contact transmission routes have been proposed to play a role. [8,11,17,57]. Although transmission was not evaluated specifically, we demonstrated that viral RNA could be detected at low levels on the fur of some ferrets but infectious virus could not be re-isolated. These findings highlight the potential importance of such indirect means of transmission, as seen in clinical settings [58]. Whilst possible virus transmission via animal fur may be of limited relevance to humans, is of particular interest given the increasing number of SARS-CoV-2 cases in farmed mink [20,22].

## 5. Conclusions

The asymptomatic transmission of SARS-CoV-2 represents a serious challenge for the control of COVID-19 [59]. Our study has demonstrated that the ferret is a suitable animal model for asymptomatic or mild SARS-CoV-2 infection in humans and other susceptible animal species. Despite the subclinical infection in ferrets, the viral shedding profile resembled that of asymptomatic human cases that are efficient in transmitting virus between individuals. In the future it will be important to evaluate the efficacy of intervention strategies in reducing the transmission of SARS-CoV-2 by asymptomatic carriers.

## Figures and Tables

**Table 1:** Study plan and sample schedule. Sampling undertaken on specific days post inoculation (dpi) ^a^ is indicated.

Dpi ^a^	Inoculation	Weight	Temperature	Nasal Wash	Throat Swab	Rectal Swab	Blood	Environmental/Fur	Necropsy
–8		x	x						
–7			x						
–6			x						
–5			x						
−4		x	x						
–3		x	x						
–2		x	x						
–1		x	x	x					
0	X (*n* = 12)	x	x			x	x	x	
1		x	x						
2		x	x	x	x	x		x	
3		x	x						2 ferrets
4		x	x	x	x	x	x	x	
5		x	x						2 ferrets
6		x	x	x	x	x		x	
7		x	x						2 ferrets
8		x	x	x	x	x	x	x	
9		x	x						
10		x	x	x	x	x		x	
11		x	x						
12		x	x						
13		x	x						
14		x	x	x	x	x	x	x	2 ferrets
15		x	x						
16		x	x						
17	Re-challenged (*n* = 2)	x	x	x	x	x		x	
18	1	x	x						
19	2	x	x	x	x	x			
20	3	x	x						
21	4	x	x	x	x	x	x	x	2 ferrets
	5								
	6								
	7	x	x	x	x	x	x	x	Re-challenged 2 ferrets

**Table 2:** Virus isolation (VI) from selected samples.

Sample (Ferret ID, dpi, sample)	VI-Positive (+) or VI-Negative (−)
**Nasal Washes**	
19243 4 dpi Nasal Wash	−
19517 4 dpi Nasal Wash	−
99873 4 dpi Nasal Wash	+
19659 4 dpi Nasal Wash	−
19675 4 dpi Nasal Wash	+
00396 4 dpi Nasal Wash	−
00479 4 dpi Nasal Wash	−
18784 4 dpi Nasal Wash	−
18988 4 dpi Nasal Wash	−
19413 4 dpi Nasal Wash	−
19517 6 dpi Nasal Wash	−
99873 6 dpi Nasal Wash	−
19659 6 dpi Nasal Wash	+
19675 6 dpi Nasal Wash	+
00479 6 dpi Nasal Wash	−
18784 6 dpi Nasal Wash	−
18988 6 dpi Nasal Wash	−
19413 6 dpi Nasal Wash	−
19517 7 dpi Nasal Wash	+
00479 7 dpi Nasal Wash	−
**Tissues**	
19517 7 dpi Soft Palate	−
19517 7 dpi Respiratory Turbinate	+
19517 7 dpi Larynx	+
19517 7 dpi Stomach	−
19517 7 dpi Oesophagus	+
00396 5 dpi Respiratory Turbinate	+
00002 3 dpi Respiratory Turbinate	−
00002 3 dpi Oesophagus	ND *
19243 5 dpi Brain	ND *

* Not determined (contaminated or toxic to cells).

## Data Availability

Source data are available upon request.

## Supplementary Materials

The following are available online at https://www.mdpi.com/1999-4915/13/1/113/s1: Figure S1. Real-time RT-qPCR quantification. Figure S2. Clinical data for daily temperature and weight monitoring.
