# Supplementary material for: Intranasal Infection of Ferrets with SARS-CoV-2 as a Model for Asymptomatic Human Infection
Source: Viruses. 2021 Jan 15;13(1):113. doi: 10.3390/v13010113 (PMC7830262; doi:10.3390/v13010113)
Supplement: Supplementary file 1 [file viruses-13-00113-s001.pdf]

Supplementary Materials: Figure S1 and S2

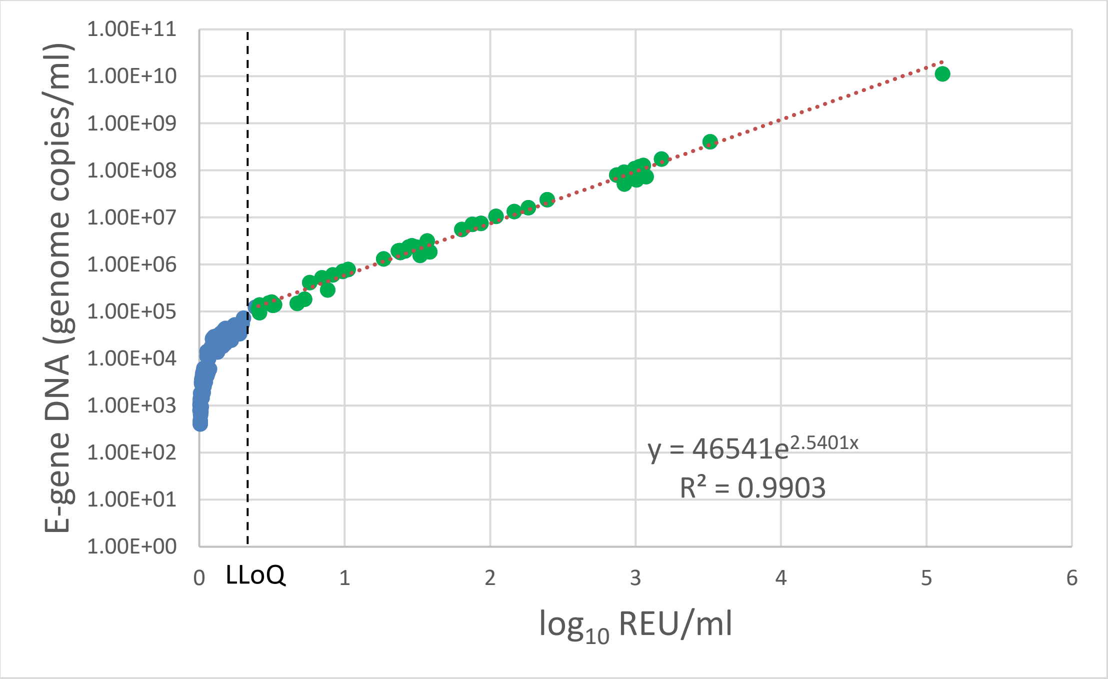

Figure S1. Real-time RT-qPCR quantification. Relative Equivalent units (REU) were calculated for a ten-fold dilution series of RNA extracted from a SARS-CoV-2/Australia/VIC01/2020 stock of known titre ($\log_{10}$ $TCID_{50}$/ml). REU values showed good correlation with the SARS-CoV-2 E gene DNA quantification standard (genome copies). The lower limit of quantification (LLoQ) for the assay is 0.4 $\log_{10}$ REU or approximately $1x10^5$ genome copies/ml. The graph shows the results of five independent assays.

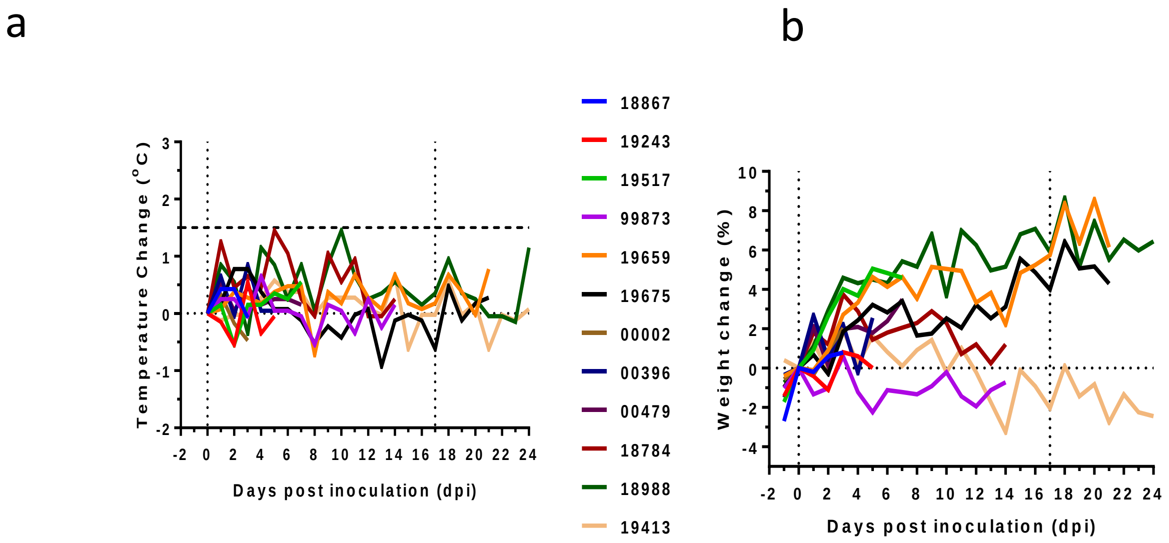

Figure S2. Clinical data for daily temperature and weight monitoring. Recorded (a) temperature (^{o}C), as an absolute change from baseline during the acclimatization period and (b) weight, as a percentage change from baseline at time of inoculation. Parameters remained within the normal range and did not exceed the mild limit for any clinical signs including temperature (+1.5^{o}C increase from baseline) or weight (10% decrease in weight from 0dpi) for the duration of the study.
